# Enhancing electric vehicle powertrain energy efficiency using robust nonlinear control approaches

**DOI:** 10.1038/s41598-025-04950-0

**Published:** 2025-06-06

**Authors:** Ilyass El Myasse, Mohamed Lmouradi, Abdelmounime El Magri, Rachid Lajouad, Pankaj Kumar

**Affiliations:** 1 LSIB Laboratory, FST Mohammedia, Hassan II University of Casablanca, 28800 Mohammedia, Morocco; 2 Jadara University Research Center, Jadara University, Irbid, Jordan; 3 EEIS Laboratory, ENSET Mohammedia, Hassan II University of Casablanca, 28800 Mohammedia, Morocco; 4 Department of Electrical and Electronics Engineering, Manipal Institute of Technology, Manipal Academy of Higher Education, Manipal, Karnataka 576104 India

**Keywords:** Electric vehicle (EV), Battery storage system, Parameter uncertainty, Robust nonlinear contro, Energy science and technology, Engineering

## Abstract

This paper addresses the issue of controlling the drivetrain of electric vehicles. Taking into account both internal and external system disturbances, including the vehicle’s mass, rotational friction of the shafts, wind speed, vehicle aerodynamics, road type, and slope constraints, the controller’s task is to ensure robustness in vehicle behavior. The significant dynamics of these disturbances and uncertainties in vehicle parameters have a substantial impact on vehicle performance. To overcome these challenges, a nonlinear model of the entire controlled system is developed. Subsequently, a robust nonlinear controller is designed using the damping function version of the backstepping design technique to compensate for all uncertain terms. Within this framework, two primary control loops are established. Firstly, a speed control loop is implemented to achieve precise tracking of the driver’s speed reference. Secondly, the machine current is optimized to generate maximum torque. A formal analysis based on Lyapunov stability is conducted to describe the control system’s performance. Despite parameter uncertainties, it is demonstrated that all control objectives are asymptotically achieved. Ultimately, all control objectives are validated through simulation results using Matlab/Simulink, showcasing the efficiency and robustness of the proposed control technique.

## Introduction

As the world moves toward more sustainable transportation, electric vehicles (EVs) are taking center stage in automotive innovation. This shift is driven by growing environmental concerns, stricter emission regulations, and rapid advancements in battery technology^1,2^. By 2040, EVs could exceed 50% of new car sales, making their role in reducing carbon emissions and improving efficiency crucial^3^. This demand calls for robust powertrain systems that adapt to various driving needs^4^. As EVs shape the future of mobility, optimizing their performance through advanced control strategies is essential.

In the domain of automotive engineering, cruise control systems play a pivotal role in enhancing driving comfort, fuel efficiency, and overall road safety^5,6^. These systems are meticulously designed to maintain a desired vehicle speed, relieving the driver from constant throttle adjustments and allowing focus on critical driving tasks^7,8^. The need for precise and effective control in such systems is paramount. While traditional proportional-integral-derivative (PID) controllers are widely adopted for their simplicity^9–11^, they often struggle with the nonlinear dynamics and parameter uncertainties (e.g., wind speed, vehicle mass) inherent in electric vehicle (EV) powertrains, requiring frequent retuning. Advanced model predictive control (MPC) offers predictive capabilities^12,13^, but its reliance on precise models and high computational demands limits real-time applicability. The literature also explores alternative structures, including fractional-order PID (FOPID)^14^, model predictive^12,13^, deep reinforcement learning^15^, fuzzy logic^16^, and real PID plus second-order derivative^17^ controllers.”

Despite the satisfactory performance often delivered by these controllers, their design process can be time-consuming and may not consistently yield optimal results. To address these challenges, researchers have embraced metaheuristic algorithms, showing promise in solving complex optimization problems^18^. In the realm of vehicle cruise control systems, the application of metaheuristic algorithms has the potential to significantly enhance the time-domain performance and stability of the control system. Consequently, this paper focuses on developing and applying an enhanced Runge Kutta (RUN) optimizer, named IRUN, to achieve superior control performance. The proposed IRUN optimizer integrates several advanced strategies, including quadratic interpolation, Laplacian segment mutation, Levy flight, and information-sharing-based local search mechanisms, to augment its effectiveness. Through the incorporation of these strategies, the resulting IRUN algorithm showcases improved optimization capabilities, making it well-suited for fine-tuning the controller.

The study focused on comprehensive modeling of the electric vehicle’s traction system, incorporating the intricate components such as the Lithium-ion battery, the Motor-Converter association, and the mechanical transmission. This modeling approach extended to encompass the vehicle’s body dynamics and longitudinal behavior. The core innovation lay in the development and application of a robust nonlinear controller, tailored to optimize the vehicle’s performance. The methodology involved meticulous design, implementation, and validation of this controller using available empirical data. The research sought to enhance understanding and efficiency in electric vehicle powertrain systems, striving to contribute to advancements in sustainable transportation technology.

In order to comprehensively evaluate the effectiveness of the proposed method, the reference speed is provided that has to press/release either the accelerator in order to reduce the error between the actual speed and the speed from a drive cycle The driver model provides the torque demand to match the drive cycle speed profile. Thereafter, from the driver set-point, the energy required to overcome the opposing forces acting on the vehicle namely Aerodynamic Drag Force,Rolling Resistance Force, represents Gradient Resistance Force and the Inertia Resistance Force, is computed. The backward approach considers a reference speed profile, as input, to determine the forces acting at the wheels and then processes backward through the powertrain.

Summarising the aforementioned literature and considering the discussion mentioned above, the main contributions of this paper can be stated as follows:

- Precision in Vehicle Speed Tracking: This paper addresses the critical challenge of ensuring that the vehicle speed accurately follows the reference speed set by the driver. This is particularly noteworthy given the various disturbances encountered, including wheel friction, wind velocity, vehicle aerodynamics, vehicle mass, and variations in road type or slope. The proposed approach focuses on mitigating these disturbances to enhance the accuracy of speed tracking, contributing to a safer and more comfortable driving experience.
- Disturbance Mitigation Strategies: Innovative strategies to mitigate disturbances, contributing to a robust, adaptable vehicle speed control system.
- Optimization of Stator Current for Maximum Torque: optimizes stator current for maximum torque, enhancing overall vehicle performance and fuel efficiency.
- Adaptability to Variable Road Conditions: Acknowledging the influence of road type and slope on vehicle dynamics, the proposed methods aim to optimize speed control in diverse road conditions. This adaptability is a key contribution, as it enhances the overall safety and stability of the vehicle across varying terrains.

These contributions have real-world benefits for EV powertrain control. Precise speed tracking improves energy efficiency and safety, whether in stop-and-go city traffic or on steep inclines, making EVs more reliable for daily drivers. Effective disturbance mitigation ensures stable performance despite external factors like wind or changing loads, reducing strain on powertrain components and extending battery life, which is key to lowering costs. Optimizing stator current for maximum torque enhances acceleration and hill-climbing, improving the driving experience and making EVs more competitive with traditional combustion vehicles. Altogether, these advancements make EV control systems more robust and adaptable, paving the way for next-generation autonomous and high-performance electric vehicles while accelerating the shift to sustainable transportation.

The rest of the paper is organized as follows: Section “Vehicle modelling” introduces the mathematic models of the whole system. In Section “Robust nonlinear controller design”, the control strategy using nonlinear robust backstepping controller is described in details. Section “System simulation” shows the simulation setup, results and analysis. Finally, concluding remarks are discussed in Section “Robustness of the proposed control”.

## Vehicle modelling

**Fig. 1:**
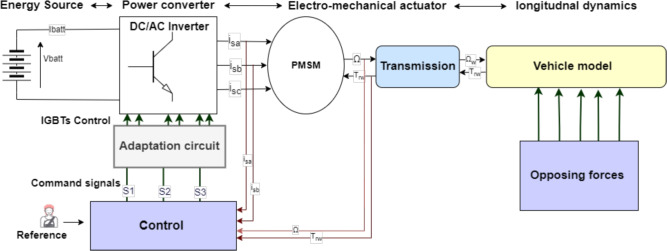
Topology of traction chain of an electric vehicle.

The considered conversion system consists, as shown in Fig. 1, of a association of several modules. On the one side, there is a storage battery is used to supply (reversible energy source) the power conversion chain through a DC/AC inverter. The role of this latter is to generate an adequate power supply to the electromechanical converter (synchronous machine), associated in turn with a transmission system representing the differential and the various mechanical connections between the traction machine and the vehicle’s wheels. On the other side, at the end of the conversion chain, there is the mechanical load where the rolling environment of the vehicle is considered (wind, slope, load, type of road, vehicle shape...).

Thanks to their better mass/power ratio and their ability to develop a much higher power level and present a more satisfactory efficiency, permanent magnet synchronous motors (PMSM) are more suitable for electric vehicles. A control unit allowing the control of all the elements of the traction chain is necessary in order to satisfy the driver requirements.

### Model of vehicle body and longitudnal dynamics

**Fig. 2:**
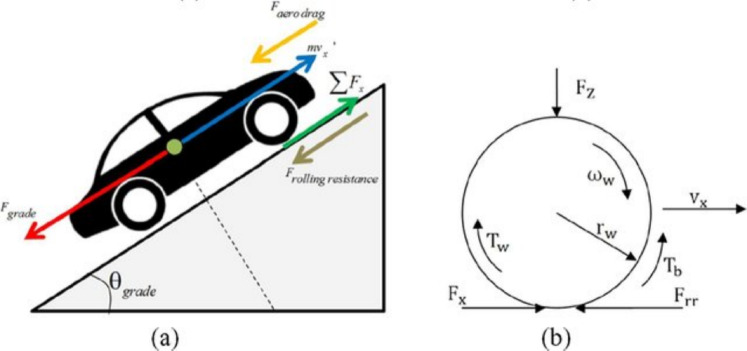
(**a**) Longitudinal vehicle dynamics model and (**b**) simplified wheel dynamics model.

An electric vehicle is subject to forces that the traction system must overcome in order to move the vehicle forward or backward, These forces are distributed as shown in Fig. 2.

Several more or less complex mechanical models are developed in the literature^19^. In this paper, the model considering the vehicle as a massive point and which undergoes a time varying forces sum has been adopted^20^. The following opposing forces are considered in the proposed model as a common technique in the literature as shown in Fig. 2:

- Gradient Resistance Force: due to the road inclination with regard to the horizontal plane.
- Rolling Resistance Force: mainly due to the friction between the tyres and the road.
- Aerodynamic Drag Force: due to the friction between the vehicle body and the air.
- Inertia Resistance Force: related to the forces required for the linear acceleration of the vehicle and the increase of the rotational speed of the rotating components

1 $$\begin{aligned} F_{tr} = F_{aero\ drag} + F_{rolling\ resistance} + F_{grade} + F_{acc} \end{aligned}$$

Where:

$F_{aero\ drag}$ denotes the Aerodynamic Drag Force, $F_{rolling\ resistance}$ designates the rolling resistance force, $F_{grade}$ represents the Gradient Resistance Force, $F_{acc}$ is the Inertia Resistance Force.

2 $$\begin{aligned} T_{r\omega }&= R_{\omega } F_{tr} \\\nonumber&= R_{\omega }\left( \frac{1}{2}\rho S_{f} C_{x}(V \pm V_{w})^2 + M g C_{rolling\ resistance} + M g \sin (\theta ) + C_{i} M \frac{dV}{dt}\right) \end{aligned}$$

Where:

$T_{r\omega }$ is the resistant torque on wheels,$R_{\omega }$ designates the wheel radius,*V* is the vehicle speed in (m/s),$V_{w}$ is the wind speed, which has a positive sign when it is tailwind and a negative sign when it is headwind,*M* is the vehicle mass in (kg),*g* is the gravitational constant $(m/s^2)$, $\theta$ is the road slope angle in radians, $\rho$ is the air density in $(kg/m^3)$,$S_f$ is the frontal surface of the vehicle, $C_x$ is the drag coefficient,$C_i$ is the inertia factor, which is assumed to be around 1.15 (a typical value),$C_{rolling\ resistance}$ is the rolling resistance coefficient.

### Model of mechanical trnasmission

In order to adjust the wheels speed $(\Omega _\omega )$ and/or torque $(T_{rm})$, a gearbox linked to the drive shaft with the wheel axis. This gearbox can be modeled by a product of reduction ratio (*r*) and efficiency representing the friction losses $(\eta _t)$^20^.

3 $$\begin{aligned} \eta _t=\dfrac{P_\omega }{P_m}=\dfrac{T_{r\omega }}{T_{rm}} \dfrac{\Omega _\omega }{\Omega } \end{aligned}$$

where $P_\omega$ output mechanical power on the wheel side, $P_m$ motor mechanical power, $T_{rm}$ motor resistive torque; $\Omega _\omega$ wheel angular velocity; $\Omega$ is the angular rotor speed.

4 $$\begin{aligned} {\left\{ \begin{array}{ll} T_{rm}=\dfrac{r}{\eta _t} T_{r\omega } \\ \Omega _\omega =r\Omega \end{array}\right. } \end{aligned}$$

where *r* reduction ratio; $\eta _t$ gearbox efficiency;

### Model of Lithium-ion battery

The modeling of batteries is an important and complex issue, i.e. a mathematical description of its chemical behavior. Indeed, it is required to model its charging and discharging behaviors according to the battery type and parameters variation i.e. state of charge (SOC), current, temperature, etc. Several battery models have been developed in previous works. A compromise between complexity, accuracy and model parameterization is considered when chosing a model : i) Thevenin-based, impedance-based, and runtime-based models. ii) Model using neural network^21^; iii) electrochemical models^22–24^; iv) Empirical models^25,26^.

**Fig. 3:**
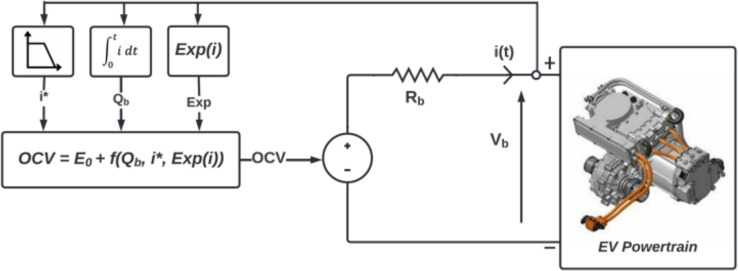
Shepherd model of the battery storage.

In the present work, the modified Shepherd model is adopted^25^. It is a dynamic model that can be parameterized to generate the charge and discharge curves. This model is based on equations that describe the battery open circuit voltage as a function of the state of charge and the exchanged current, as shown in Fig. 3.

The open circuit voltage (OCV) in charge and discharge modes of a Lithium-ion battery, according to the shpherd model, is described in(5) (if $i<0$) and (6) (if $i>0$) respectively (see (7)(8)).

- Charge mode $$(i<0)$$
  5 $$\begin{aligned} OCV=&E_0-K_i^*\dfrac{Q_n}{0.1Q_n-Q_b}+KQ_b\dfrac{Q_n}{0.1Q_n-Q_b}+A\exp (-BQ_b) \end{aligned}$$
- Discharge mode $$(i>0)$$
  6 $$\begin{aligned} OCV=&E_0-K_i^*\dfrac{Q_n}{Q_n-Q_b}+KQ_b\dfrac{Q_n}{0.1Q_n-Q_b}+A\exp (-BQ_b) \end{aligned}$$

where $E_0$ is the constant voltage; $Q_n$ is the nominal capacity; $Q_b$ is the discharged capacity; *K* is the polarization constant; *A* is the amplitude of the exponential zone; *B* is the time constant inverse of the exponential zone; and $i^*$ is filtred current with low dynamic. Several methods, for estimating the battery’s SoC, exist in the literature. These methods are mainly based on the electrical quantity’s measurement (voltage and current) and battery parameters. According to Shepherd model, assume that the initial SoC is known and the battery current is available. The simplest and robust method named Coulomb counting method (CCM) can be used. The Eqs. (7) and (8) allow to compute *SoC*(*t*) based on the battery current measurement *i*(*t*).

7 $$\begin{aligned} & Q_b(t)=\dfrac{1}{3600}\int _{0}^t i(t)dt \end{aligned}$$

8 $$\begin{aligned} & SoC(t)=SoC_0-100\dfrac{Q_b}{Q_n} \end{aligned}$$

The battery voltage $V_b$ depends on the current direction and can be described as follows :

9 $$\begin{aligned} V_b(t)=OCV(t)\pm R_bi(t) \end{aligned}$$

**Fig. 4:**
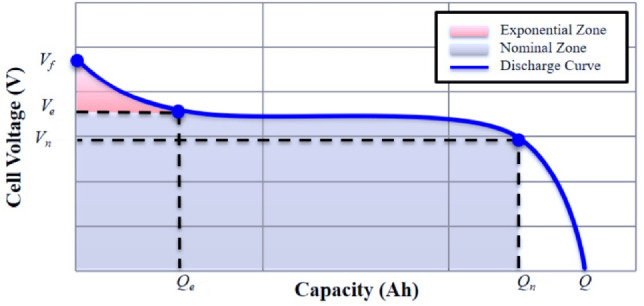
Discharge curve of a Li-ion battery.

Generally, the battery parameters quoted in (5), (6) and (9), namely $R_b$ which is the internal resistance of the battery K, A, B and $E_0$ can be determined empirically by OCV$(Q_b)$ curve especially from the discharge curve or from the datasheet provided by the battery manufacturer^25,27^. Figure 4 shows the characteristic of voltage variation as a function of charge capacity during a discharge case^28^. It is a non-linear characteristic that allows the battery-parameters to be determined as follows:

10 $$\begin{aligned} A&=V_f-V_e \end{aligned}$$

11 $$\begin{aligned} B&=\dfrac{\alpha }{Q_e} \end{aligned}$$

12 $$\begin{aligned} K&=\beta [V_f-V_n+A(\exp (-BQ_n)-1)]\dfrac{Q_f-Q_n}{Q_n} \end{aligned}$$

13 $$\begin{aligned} E_0&=V_f+K+R_ii_n-A \end{aligned}$$

14 $$\begin{aligned} R_b&=V_n\dfrac{1-\eta }{0.2Q_n} \end{aligned}$$

where $V_f$ is the fully charged voltage; $V_e$ the voltage at the end of exponential zone; $V_n$ is the rated voltage; $Q_n$ is the maximum capacity of the battery; $Q_e$ is the capacity at the end of exponential zone; $i_n$ is the rated current of the battery; $\eta$ represents the battery efficiency; $\alpha$ and $\beta$ constants values are usually determined to improve the fit to a battery data^26^. *

### Model of the motor-converter association

**Fig. 5:**
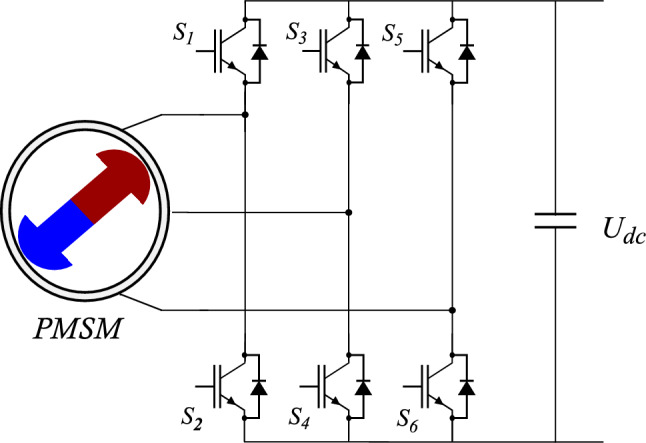
Electrical scheme of a PMSM with rectifier.

The structure of the sub-systém considered is described by the Fig. 5. It consist of converting electric energy provided by the battery into mechanical energy, using the permanent magnet synchronous motors (PMSM)^29^. After the well-known Park transformation, the mathematical model of the converter-machine association in the dq frame (more suitable for developing control laws), can be described by the Eqs. (15a)–(15c)^30^.

15a $$\begin{aligned} \dfrac{d\Omega }{dt}&= \dfrac{3}{2} \dfrac{k_m}{J_m}i_q-\dfrac{F_{m}}{J_m}\Omega -\dfrac{T_{rm}}{J_m} \end{aligned}$$

15b $$\begin{aligned} \dfrac{di_{q}}{dt}&= -\dfrac{R_s}{L_s}i_{q}-p\Omega i_{d}-\dfrac{k_m}{L_s} \Omega + \dfrac{u_{q}}{L_{s}}V_{b} \end{aligned}$$

15c $$\begin{aligned} \dfrac{di_{d}}{dt}&= -\dfrac{R_s}{L_s}i_{d} +p\Omega i_{q} + \dfrac{u_{d}}{L_{s}}V_{b} \end{aligned}$$

where ${R_s}$ and ${L_s}$ are the stator resistor and inductance; ${i_d}$ and ${i_q}$ denotes the stator currents in dq coordinates; ${J_m}$, ${F_m}$ and p are respectively the rotor inertia, viscous coefficient and number of poles pairs; $T_{rm}$ denote the resistant torque on the motor which include all forces applied on the vehicle ( see Eq. (4) and (2)); ${k_m=p.\phi _r}$ is the motor coefficient, with $\phi _r$ is the rotor flux norm; $u_d$ and $u_q$ are the DC/AC input control in the dq coordinates; $V_b$ is the battery voltage described in (9).

In view from the motor axis, the resistant torque $T_{rm}$ take the following expression, obtained by replacing the Eq. (2) in (2.2).

16 $$\begin{aligned} T_{rm}=K_{D\omega }(V \pm V_{w})^2+\delta _{DM} M+ k_i M \dfrac{d\Omega }{dt} \end{aligned}$$

where $K_{D\omega }$ is the wind disturbance coeficient, $\delta _{DM}$ is the mass disturbance function, which take variation following the state of the road and the slope percentage, $K_i$ is the inertia coeficient.

17a $$\begin{aligned} K_{D\omega }&= \dfrac{rR_\omega }{2\eta _t}\rho S_f C_x \end{aligned}$$

17b $$\begin{aligned} \delta _{DM}&= \dfrac{rR\omega }{\eta _t}g(C_{roll}+S_{\%} ) \end{aligned}$$

17c $$\begin{aligned} K_i&= \dfrac{C_i(rR_\omega )^2}{\eta _t} \end{aligned}$$

####  Remarks

- $\delta _{DM}$ is very dependent on the mass of the vehicle that its variation is generally very limited. In this work, we consider the parameters of the function $\delta _{DM}$ as constant and defined by their average values.
- As the dynamics of the aerodynamic force is slowly with respect to the speed, one can approximate $F_{aer}$ to a linear function around the rated speed. Using Taylor young’s theorem, the aerodynamic torque takes the following expression:

18 $$\begin{aligned} T_{ae}&= K_{D\omega }(V \pm V_{w})^2 \\\nonumber&= A_4 + (A_3V_\omega +A_2)\Omega +A_1(V_\omega )^2 \end{aligned}$$

where $A_i,(i=1,..4$) are a constants coefficients depending to the vehicle parameters, as defined bellow:

19a $$\begin{aligned} A_1&= K_{D\omega }=\dfrac{rR_\omega }{2\eta _t}\rho S_f C_x \end{aligned}$$

19b $$\begin{aligned} A_2&= \dfrac{(rR_\omega )^3}{\eta _t}\rho S_f C_x \Omega _0 \end{aligned}$$

19c $$\begin{aligned} A_3&=\dfrac{(rR_\omega )^2}{\eta _t}\rho S_f C_x \end{aligned}$$

19d $$\begin{aligned} A_4&= \dfrac{(rR_\omega )^3}{2\eta _t}\rho S_f C_x \Omega _0^2 \end{aligned}$$

Using (16) and (18) it follows from (15) that the speed dynamics undergoes the Eq. (20), defined in the follo state space representation of the whole system including the vehicle environment, mechanical transmission and motor-converter association.

20 $$\begin{aligned} \dfrac{d\Omega }{dt} = \dfrac{3}{2} \dfrac{k_m}{J} i_q -\dfrac{F_v}{J} M - \dfrac{\psi _w}{J} \end{aligned}$$

where *J* is the whole system inertia $J=J_m+K_i M$, $F_v$ is the vehicle friction including the motor friction, transmission friction, and the linear terme of wind friction by the vehicle, which is expressed by $F_v=F_m+A_3V_\omega +A_2$ and $\psi _w$ is the non linear influence of wind velocity on vehicle taking the expression $\psi _w=A_1V_\omega ^2+A_4$. Now, let us introduce the state variables $x_1=\Omega$, $x_2=i_q$ and $x_3=i_d$ and the inputs control $u_1=u_q$ and $u_2=u_d$. Then the state space representation of the traction system can be presented as:

21a $$\begin{aligned} \dfrac{dx_1}{dt}&= \dfrac{3}{2} \dfrac{k_m}{J}x_2-\dfrac{F_v}{J}x_1-\dfrac{\delta _{DM}}{J}M-\dfrac{\psi _w}{J} \end{aligned}$$

21b $$\begin{aligned} \dfrac{dx_2}{dt}&= -\dfrac{R_s}{L_s}x_2-px_1 x_3-\dfrac{k_m}{L_q}x_1 + \dfrac{V_b}{L_{s}}u_1 \end{aligned}$$

21c $$\begin{aligned} \dfrac{dx_3}{dt}&= -\dfrac{R_s}{L_s}x_3+px_1 x_2 + \dfrac{V_b}{L_{s}}u_2 \end{aligned}$$

## Robust nonlinear controller design

### Control objectives

This section focuses on developing a nonlinear controller which is capable of achieving the following operational control objectives:

- Speed regulation : the vehicle speed must track, as accurately as possible the reference speed specified by the driver, despite the several disturbance, in particular the wheel friction, wind velocity, vehicle aerodynamic, vehicle mass and road type or slope inconvenience.
- Regulate the d-axis current component to zero, in order to optimize the stator current in the machine and thus develop a maximum torque^31^.

In the following subsections, a nonlinear state feedback controller will be performed using the Robust Backstepping technique to achieve the above control objectives. The choice of backstepping technique in this study is motivated by its systematic, recursive design process. Backstepping allows for step-by-step stabilization of subsystems, such as speed and current loops, while explicitly addressing nonlinearities and uncertainties through Lyapunov-based stability guarantees^32,33^.

### Vehicle model with parameter uncertainties

The disturbance undergoes by the vehicule are not supposed to be known $(F_v, V_\omega )$ or not accuratly mesurable (M). It’s just supposed to be delimited within a known intervals as shown below:

22a $$\begin{aligned} M&=M_0(1+\Delta _M) \end{aligned}$$

22b $$\begin{aligned} F_v&=F_{v0}(1+\Delta _F) \end{aligned}$$

22c $$\begin{aligned} V_\omega&=V_{\omega 0}(1+\Delta _\omega ) \end{aligned}$$

with $M_0$ the nominal mass of the vehicle, $F_{v0}$ and $V_{\omega 0}$ are respectively the mean values of the vehicle friction and wind velocity, concerning $\Delta _M$, $\Delta _F$ and $\Delta _\omega$ denotes the possibly varying uncertainties such that.

23a $$\begin{aligned} \Delta _M^{MIN}&\le \Delta _M \le \Delta _M^{MAX} \end{aligned}$$

23b $$\begin{aligned} \Delta _F^{MIN}&\le \Delta _F\le \Delta _F^{MAX} \end{aligned}$$

23c $$\begin{aligned} \Delta _\omega ^{MIN}&\le \Delta _\omega \le \Delta _\omega ^{MAX} \end{aligned}$$

where $\Delta ^{MIN}$ and $\Delta ^{MAX}$ are known bounds, using (22) in (21a), the dynamics of the speed is described as follows:

24 $$\begin{aligned} \dfrac{dx_1}{dt} = \dfrac{3}{2} \dfrac{k_m}{J}x_2-\dfrac{F_{v0}}{J}x_1-\dfrac{\delta _{DM}}{J}M_0-\dfrac{\psi _w}{J}+\phi _1\Delta \end{aligned}$$

With $\phi _1$ and $\Delta$ are the uncertain terms defined as:

25 $$\begin{aligned} \phi _1&=[ x_1 \quad \delta _{DM} \quad K_{\omega 1} \quad K_{\omega 2}] \nonumber \\ \Delta ^T&=-\dfrac{1}{J}[F_{V0}\Delta _{F}\quad M_{0}\Delta _{M} \quad V^2_{\omega 0}\Delta _{\omega } \quad (V_{\omega 0}\Delta _{\omega })^2 ] \nonumber \\ \Delta ^T&=[\Delta _1 \quad \Delta _2 \quad \Delta _3 \quad \Delta _4] \end{aligned}$$

### Speed regulation loop

Now, the goal is to designe the control law of the vehicule speed reference tracking, which is foist by the driver. Basing on robust backstepping technique, where the machine parameters and the vehicle disturbances must be tolerated, $u_1$ in (21b) stand as input control of the loop, so let consider the following speed tracking error.

26 $$\begin{aligned} z_1=x_1-\Omega _{ref} \end{aligned}$$

By substituting (24) in (26), the error dynamics can be described as follows:

27 $$\begin{aligned} \dot{z_1} = \dfrac{3}{2} \dfrac{k_m}{J}x_2-\dfrac{F_{v0}}{J}x_1-\dfrac{\delta _{DM}}{J}M_0-\dfrac{\psi _w}{J}+\phi _1\Delta - \dot{\Omega _{ref}} \end{aligned}$$

To get stabilizing the subsystem (24), let consider $\alpha =\dfrac{3}{2} \dfrac{k_m}{J}x_2$ as a virtual control input for $z_1$ dynamics, which must track to the stabilizing function $\alpha _{ref}$ defined as mentioned below.

28 $$\begin{aligned} \alpha _{ref}=-\varsigma _1z_1-k_1|\phi _1|^2z_1+\dfrac{F_{v0}}{J}x_1-\dfrac{\delta _{DM}}{J}M_0+\dfrac{\psi _{w0}}{J} + \dot{\Omega }_{ref} \end{aligned}$$

Note that $|\phi _1|$ denotes the Euclidean norm of $\phi _1$ and $\varsigma _1>0$ is a negative scalar design parameter. The quantity $k_1|\phi _1|^2z_1$ is a nonlinear damping term that is introduced to dominate the unknown term $\phi _1\Delta$.

If $\alpha =\alpha _{ref}$, one would have:

29 $$\begin{aligned} \dot{z_1} =-\varsigma _1z_1-(k_1|\phi _1|^2z_1-\phi _1\Delta ) \end{aligned}$$

Then, if $V_1=0.5z_1^2$ considered as the the Lyapunov function candidate, one would get the following time-derivative along the $z_1$ -trajectory.

30 $$\begin{aligned} \dot{V}_1&=z_1\dot{z_1}=-\varsigma _1z_1^2-(k_1|\phi _1|^2z_1-\phi _1\Delta )z_1 \end{aligned}$$

Note that in case of no variation in disturbances $(\Delta =0)$, the dynamic of the Lyapunov function (30) is defined as negative function $(\dot{V}_1<0)$; consequently the $z_1$ error vanish in a finite time. However, to ensure regulation in all cases, let introduce a new error $z_2=\alpha -\alpha _{ref}$. The virtual control input (28) should track $\alpha _{ref}$, in order to ensure global loop regulation and eliminate the associated error $z_1$. Nevertheless, using the fact that $\alpha =z_2+\alpha _{ref}$ in (27), it follows that the $z_1$-dynamics undergos the following equation:

31 $$\begin{aligned} \dot{z_1} =-\varsigma _1z_1-(k_1|\phi _1|^2z_1-\phi _1\Delta )+z_2 \end{aligned}$$

The next aim is to ensure the asymptotic stability of the subsystem (21a)-(21b); i.e. the two tracking errors $(z_1,z_2)$ must converge to zero in a finite time. Thus, the time derivative of$z_2$-error gives

32 $$\begin{aligned} \dot{z_2} =&\beta (x)-\big (\varsigma _1+(k_1|\phi _1|^2)^2z_1+(\varsigma _1+k_1|\phi _1|^2)^2z_2\big )+\phi _2\Delta -\ddot{\alpha }_{ref}+\gamma (x)u_1 \end{aligned}$$

with

33 $$\begin{aligned} \beta (x)&=-\dfrac{3}{2} \dfrac{k_m}{J}\Big (\dfrac{R_s}{L_s}x_2+px_1 x_3+\dfrac{k_m}{L_s}x_1\Big )+\Big (\dfrac{F_{v0}}{J}-2k_1x_1z_1\Big )\nonumber \\&\Big (-\dfrac{F_{v0}}{J}x_1+\dfrac{3}{2}\dfrac{k_m}{J}x_2-\dfrac{\delta _{DM}}{J}M_0-\dfrac{\psi _{\omega 0}}{J}\Big ) \end{aligned}$$

34 $$\begin{aligned} \phi _2&=\phi _1\Big (\dfrac{F_{v0}}{J}+(\varsigma _1+k_1|\phi _1|^2)+2k_1x_1z_1\Big ) \end{aligned}$$

35 $$\begin{aligned} \gamma (x)&=\dfrac{3}{2} \dfrac{k_m}{J}\dfrac{V_b}{L_s} \end{aligned}$$

To recap, the errors dynamics $\dot{z_1}$ and $\dot{z_2}$ finally follow up the equations rewritten below:

36 $$\begin{aligned} \dot{z_1}&=-(\varsigma _1+k_1|\phi _1|^2)z_1-\phi _1\Delta +z_2 \end{aligned}$$

37 $$\begin{aligned} \dot{z_2}&=\beta (x)-(\varsigma _1+k_1|\phi _1|^2)^2z_1+(\varsigma _1+k_1|\phi _1|^2)z_2+\phi _2\Delta -\ddot{\Omega }_{ref}+\gamma (x)u_1 \end{aligned}$$

To get the stabilizing control law for the latter errors dynamics an augmented candidate Lyapunov function is defined as:

38 $$\begin{aligned} V_2=0.5z_1^2+0.5z_2^2 \end{aligned}$$

and using (36)-(37), the time derivative of $V_2$ yields:

39 $$\begin{aligned} \dot{V_2}=-(\varsigma _1+k_1|\phi _1|^2)z_1+ \phi _1\Delta z_1+z_1z_2+z_2\dot{z_2} \end{aligned}$$

Substituting the Eq. (37) in (39) turns out to be:

40 $$\begin{aligned} \begin{aligned} \dot{V_2}&=-(\varsigma _1+k_1|\phi _1|^2)z_1^2+(\phi _1z_1+\phi _2z_2)\Delta +z_1z_2 \\&\quad +\Big (\beta (x)-(\varsigma _1+k_1|\phi _1|^2)^2z_1+(\varsigma _1+k_1|\phi _1|^2)z_2-\ddot{\alpha }_{ref}+\gamma (x)u_1\Big )z_2 \end{aligned} \end{aligned}$$

According to (40), to ensure the convergence of the system in a defined time, the following control law $u_1$ is proposed.

41 $$\begin{aligned} \begin{aligned} u_1&=\gamma (x)^{-1}(-z_1-\beta (x)+(\varsigma _1+k_1|\phi _1|^2)^2z_1-(\varsigma _1+k_1|\phi _1|^2)z_2-(\varsigma _2+k_2|\phi _2|^2)^2z_2+\ddot{\Omega }_{ref} \end{aligned} \end{aligned}$$

where $\varsigma _2>0$ is a new positive design parameter for the second loop, and $k_2 |\phi _2|^2 z_2$ is an introduced additional nonlinear damping term to overcome the second uncertain term $\phi _2 \Delta$. Considering (41), the time derivative of the Lyapunov function $\dot{V_2}$ becomes:

42 $$\begin{aligned} \dot{V_2}=-\varsigma _1{z_1}^2-\varsigma _2{z_2}^2-k_1|\phi _1|^2{z_1}^2-k_2|\phi _2|^2{z_2}^2+(\phi _1z_1+\phi _2z_2)\Delta \end{aligned}$$

As can be easily deduced from (42), $\dot{V_1}$ is defined as negative function $(\dot{V_1}<0)$ although the uncertain term varies or disappears $(\Delta =0)$. The stability analysis of the overall system (21a)-(21c) will be summarized in Theorem 1. Now, one determines the remaining control input $u_2$.

### d-Axis current regulation

Now the target is the control of d-axis current $x_3$ whose dynamics is given by the Eq. (21c). For this let’s consider the tracking error $z_3=x_3-i_{dref}$. Then, by time derivative of $z_3$ and using (21c), one obtains the error dynamics.

43 $$\begin{aligned} \dot{z_3} = -\dfrac{R_s}{L_s}x_3+px_1 x_2 + \dfrac{V_b}{L_{s}}u_2- i_{dref} \end{aligned}$$

To achieve objective (ii) and make $x_3$ converges asymptotically to $i_{dref}=0$, let’s introduce a new quadratic Lyapunov function $V_3=0.5z_3^2$. The asymptotic stability is provided when $\dot{V_3}$ is negative definite, given in (44).

44 $$\begin{aligned} \dot{V_3} = \left(-\dfrac{R_s}{L_s}x_3+px_1 x_2 + \dfrac{V_b}{L_{s}}u_2 \right)z_3=-\varsigma _3{z_3}^2 \end{aligned}$$

Where $\varsigma _3>0$ is a new design parameter. Now, the input control of the d-axis loop can be easily deduced from (44) as follows:

45 $$\begin{aligned} u_2 =\dfrac{L_{s}}{V_b} \left(\dfrac{R_s}{L_s}x_3-px_1 x_2-\varsigma _3z_3 \right) \end{aligned}$$

Finally combining (45) and (43) gives:

46 $$\begin{aligned} \dot{z_3} =-\varsigma _3z_3 \end{aligned}$$

To finish the design of the controller, in the following theorem, the control closed loops are analysed.

#### Theorem 1

Consider the traction chain system described by the nonlinear model (21a)-(21b) and the control laws (28), (41) and (45), there exist a Lyapunov function candidate $$V=V_2+V_3$$ and a positive constants $$\varsigma _i$$ ,$$(i=1,2,3)$$and $$(k_1$$,$$k_2)$$ such that:

- The resulting closed-loop system undergoes, in the $(z_1, z_2, z_3)$-coordinates, the following equation:
  47a $$\begin{aligned} \dot{z_1}&=-(\varsigma _1+k_1|\phi _1|^2)z_1-\phi _1\Delta +z_2 \end{aligned}$$
  47b $$\begin{aligned} \dot{z_2}&=-(\varsigma _2+k_2|\phi _2|^2)z_2-\phi _2\Delta \end{aligned}$$
  47c $$\begin{aligned} \dot{z_3}&=-\varsigma _3z_3 \end{aligned}$$
  with $$(z_1=x_1-\Omega _{ref}), (z_2=\alpha -\alpha _{ref}),$$ and $$(z_3= x_3-i_{dref})$$
- The error vector $z(t)=(z_1,z_2,z_3)$ converges exponentially to a compact neighborhood of the origin [0 0 0]. Consequently, whatever the initial conditions, the observation error *z*(*t*) can be made arbitrarily small letting $(k_1,k_2)$ be sufficiently larges.

#### Proof

(i). Equation (47) is specifically obtained by substituting (36) into (37) and from Eqs. (27) and (46).
(ii). To prove the second part (ii), let us consider the Lyapunov candidate function :
  48 $$\begin{aligned} V=V_2+V_3=0.5(z^2_1+z^2_2+z^2_3) \end{aligned}$$
  It easily follows that :
  49 $$\begin{aligned} \dot{V}=&-\Big (\varsigma _1z^2_1+\varsigma _2z^2_2+\varsigma _3z^2_3+(k_1|\phi _1|^2)z^2_1+\phi _1\Delta z_1)+(k_2|\phi _2|^2)z^2_2+\phi _2\Delta z_2)-z_1z_2\Big ) \end{aligned}$$
  $\dot{V}$ can be bounded as follows:
  50 $$\begin{aligned}&\dot{V}<-\left(\varsigma _1z^2_1+\varsigma_2z^2_2+\varsigma _3z^2_3-|z_1||z_2|+k_1 \left(|\phi_1||z_1|-\dfrac{||\Delta ||_\infty }{2k_1}\right)\right.\\&\qquad\left. +k_2 \left(|\phi_2||z_2|-\dfrac{||\Delta ||_\infty }{2k_2}\right)-\dfrac{||\Delta||_\infty }{4k_1}-\dfrac{||\Delta ||_\infty }{4k_2}\right)\end{aligned}$$
  The inequality (50) can be rewritten under the following form :
  51 $$\begin{aligned} \dot{V} \le -\varsigma V +\dfrac{||\Delta ||_\infty }{2\varsigma k} \end{aligned}$$
  where $||\Delta ||_\infty$ denotes the $L_\infty$ norm and $\varsigma = \min (\varsigma _1, \varsigma _2, \varsigma _3)$ and $k = \min (k_1, k_2)$. Recalling that $V=0.5(z_1^2+z_2^2+z_3^2)$, which implies that the vector $(z_1 (t)$,$z_2 (t)$ and $z_3 (t))$ converges exponentially to the following compact:
  52 $$\begin{aligned} z^2_1+z^2_2+z^2_3 \le \dfrac{||\Delta ||_\infty }{\varsigma k} \end{aligned}$$
  It is clear that the dimensions of such a compact are inversely proportional to $\varsigma k$.

$$\square$$

## System simulation

### Protocol of simulation

**Fig. 6:**
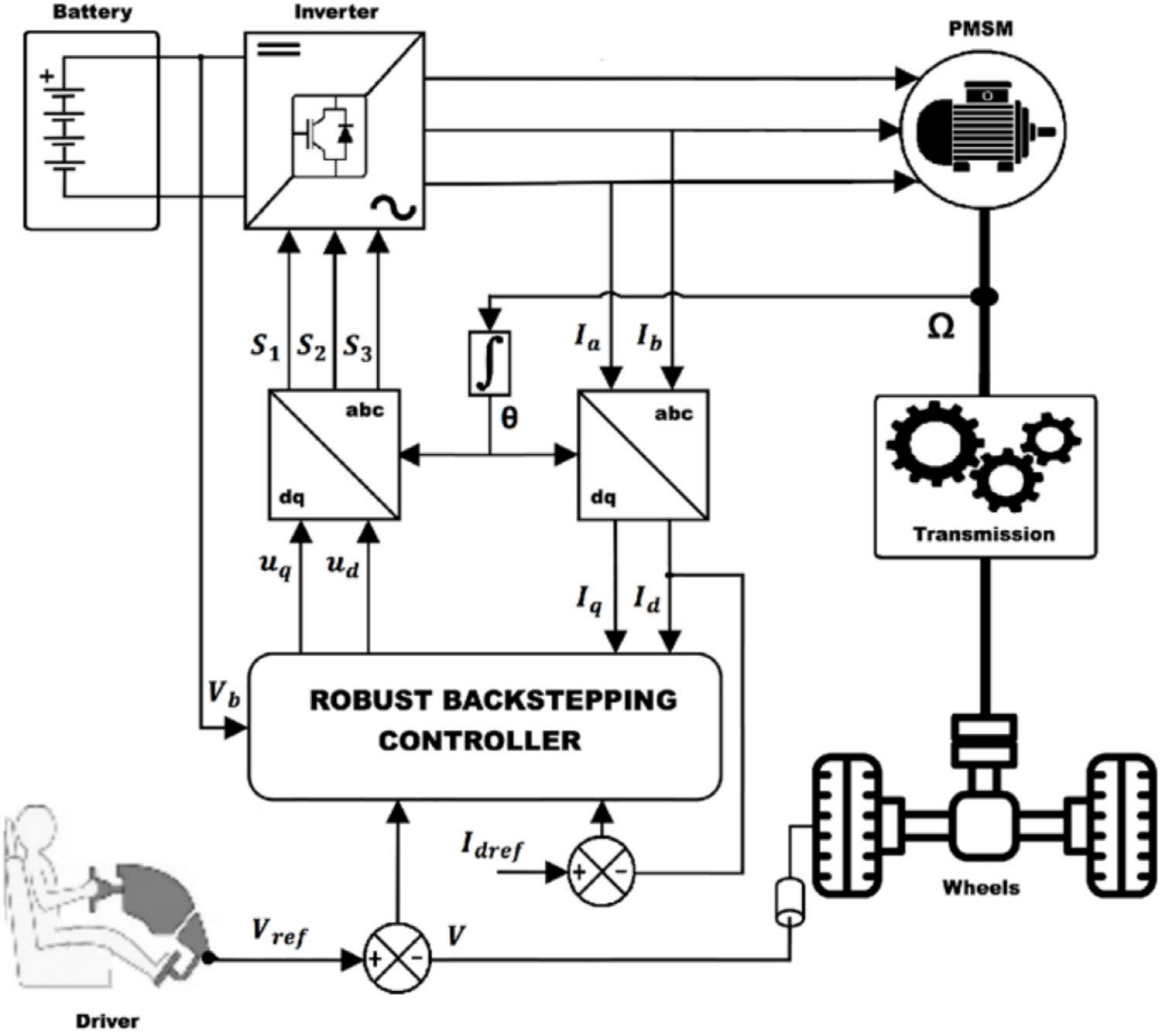
System simulation protocol.

In this section, investigates the performance of the proposed controller the module of the EV diagrams described by Fig. 6 is established in the MATLAB/Simulink environment.

**Fig. 7:**
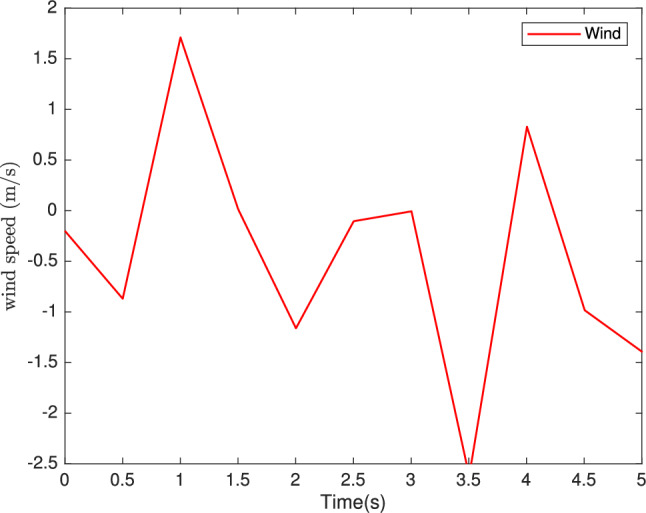
Wind speed (m/s).

**Fig. 8:**
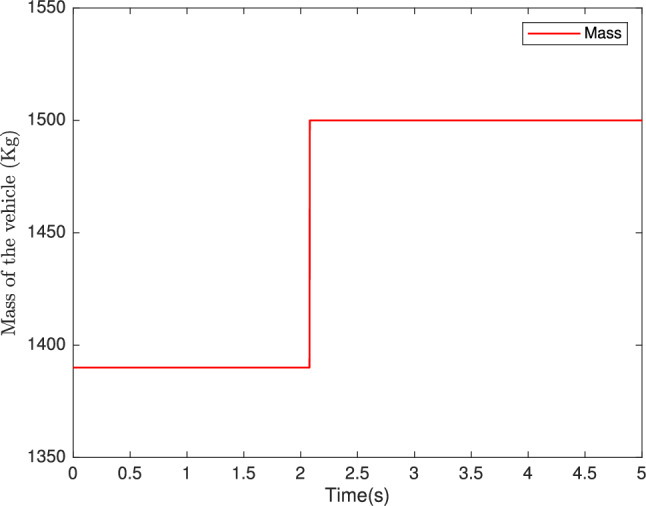
Mass of vehicle (Kg).

To evaluate the performance of the proposed controllers, we take the wind speed profile shown in Fig. 7, and the vehicle mass shown in Fig. 8 and select a profile over a wide range of variation of the longitudinal dynamics of the vehicle, The simulation parameters are given in Table 1.

#### Remark 1

The control parameters of the robust backstepping controller were tuned using a trial-and-error method in MATLAB/Simulink to achieve optimal performance. This iterative process aimed to minimize key metrics such as convergence time and steady-state error. While trial-and-error proved effective for this study, alternative optimization methods could be explored for more systematic parameter tuning, such as trajectory and velocity planning using quartic bezier curves^34^ or QPSOMPC-based chassis coordination control^35^, which provide advanced optimization frameworks for vehicle control applications.

**Table 1:** VE system characteristics^36^.

Vehicle body		
Characteristic	Values	Units
Curb weight	1390	kg
Aerodynamic drag coeff	0.3	-
Frontal area	2.38	m$^2$
Wheelbase	2570	mm

Powertrain		
Characteristic	Values	Units
Motor type	PMSM	-
Rated speed	1500	rpm
Torque sensitivity	0.6324	N.m/A
No. of pole pairs	4	–
Stator resistance	0.916	$m\Omega$/phase
D-axis inductance Ld	3.55	mH/phase
Q-axis inductance Lq	3.55	mH/phase
BEMF constant ($\lambda$)	0.1054	V/elect rad/s
Friction coefficient (F)	0.001871	Nm/rad/s
Moment of inertia (motor and load) (J)	0.00243	kg.m2

Transmission		
Simple fixed gear ratio	1:1	–

### Simulation results

**Fig. 9:**
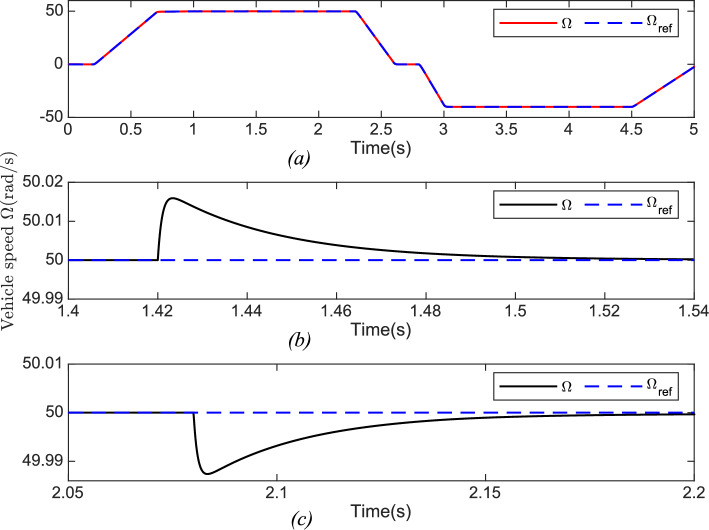
**Top:** Vehicle speed $\Omega (rad/s)$ and its Reference $\Omega _{ref} (rad/s)$. **Bottom:** Zoom on Vehicle speed *rad*/*s*.

Use of the robust backstepping control technique, has demonstrated remarkable resilience to variations in the vehicle’s longitudinal dynamics. Figure 9a vividly illustrates the speed dynamics, demonstrating exceptional responsiveness with a very fast response time. Figures 9b and c offer zoomed views to show the response of the proposed control to rapid changes in the speed reference signal. This is a key achievement, as it signifies that the speed of the electric vehicle adeptly adheres to the reference trajectory.

**Fig. 10:**
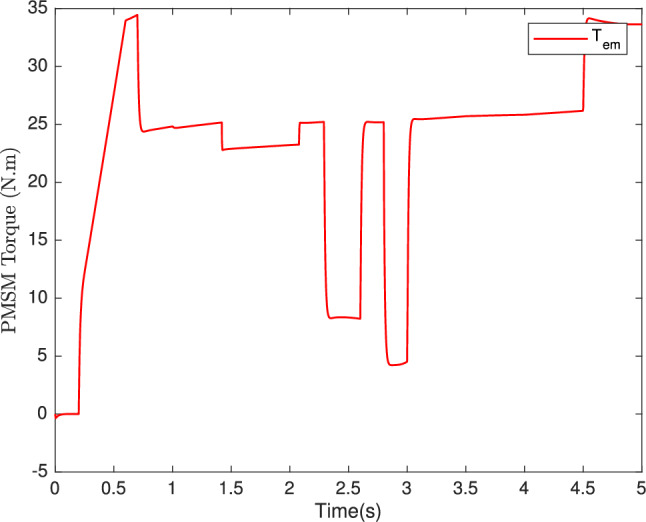
Torque motor.

**Fig. 11:**
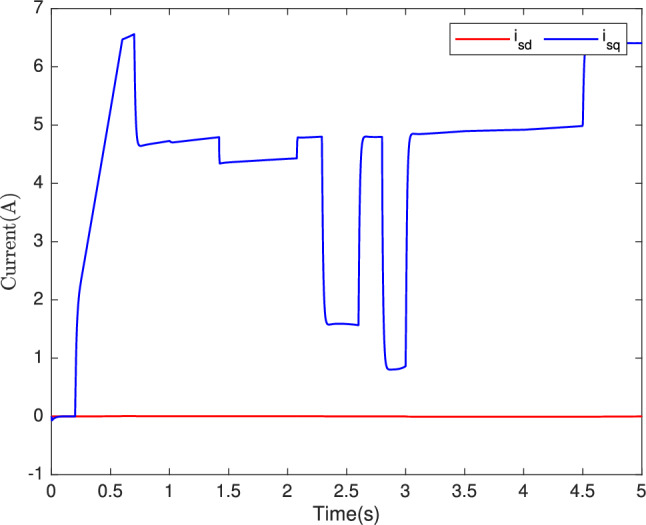
Stator current $$i_{sd}, i_{sq} (A)$$.

**Fig. 12:**
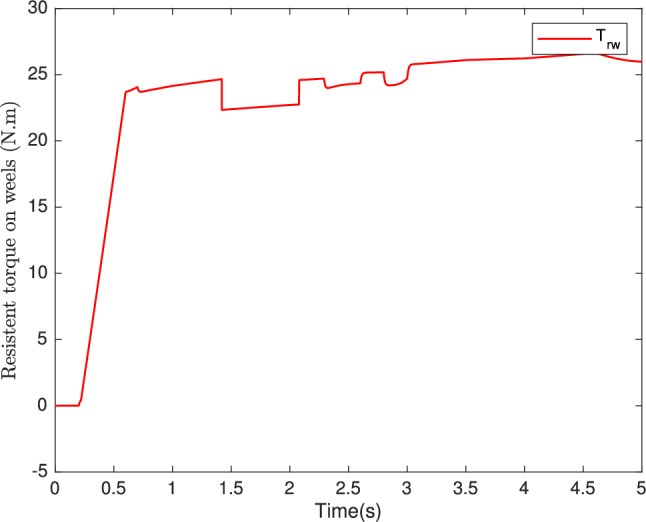
Resistant torque on wheels (N.m).

**Fig. 13:**
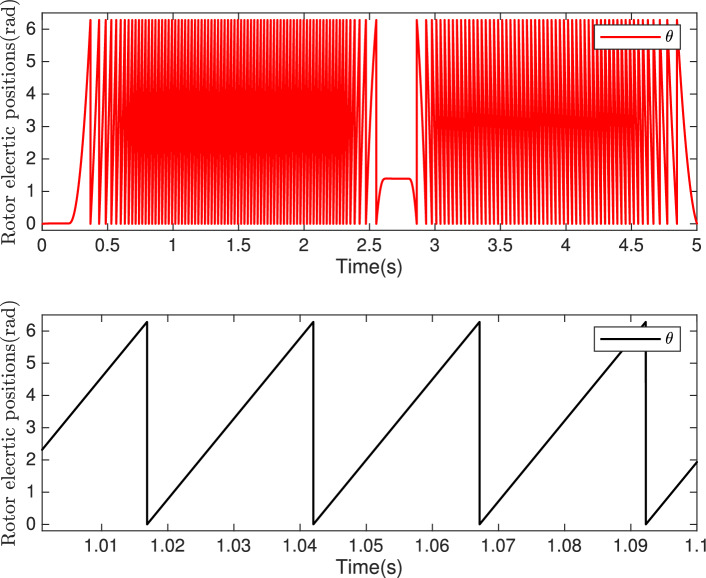
**Top:** the electrical angle $\theta$. **Bottom:** Zoom on the electrical angle $\theta$.

**Fig. 14:**
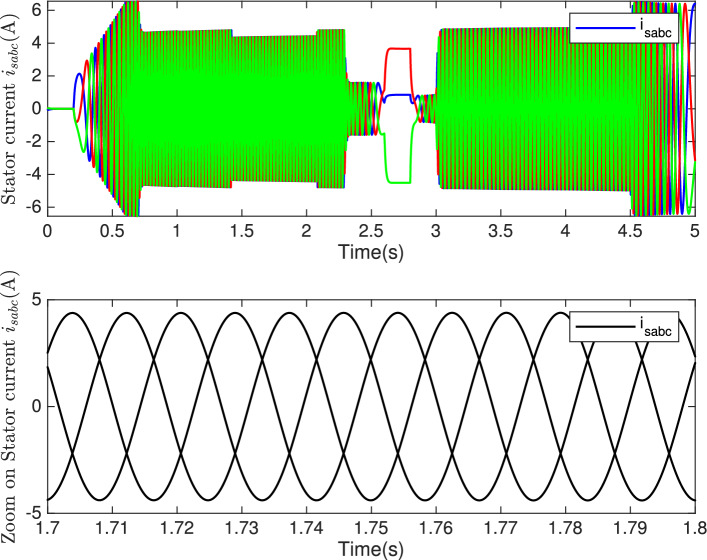
**Top:** Stator current $i_{sabc}$ (A). **Bottom:** Zoom on stator current.

In Fig. 10, we can observe the torque response, showcasing the adaptability of our control system to variations in parameters impacting longitudinal dynamics. The ability of torque to fluctuate appropriately in response to changes in vehicle mass, friction, and wind speed further exemplifies the system’s robustness. Moreover, Fig. 11 provides a snapshot of another essential control objective. Here, the $i_{sq}$ current, which represents the machine’s torque, exhibits a commendable performance. Notably, the $i_{sd}$ current remains consistently at zero, underscoring the effectiveness of our control strategy in both static and dynamic current responses. In sum, our control system, based on the backstepping robust control technique, excels in maintaining high-speed dynamics with a short response time, as demonstrated in Fig. 9, the speed tracking performance is indeed impressive. Figures 10 and 12 showcase the system’s adaptability, and Fig. 11 attests to the successful management of static and dynamic current responses. These results affirm the robustness and practicality of our approach, which holds promise for real-world applications.

Figure 13 demonstrates the behavior of the electrical angle $\theta$ over time, indicating precise and stable control as highlighted at the bottom of this figure. The three-phase stator currents ($i_{sabc}$) are shown in Fig. 14. The zoomed-in region displays near-perfect sinusoidal waveforms for the stator currents, confirming harmonic integrity and minimal distortion during steady-state operation. The system demonstrates precise and reliable tracking of both the electrical position $\theta$ and the stator currents ($i_{sabc}$) , maintaining stability and accuracy even during dynamic operating conditions.

## Robustness of the proposed control

**Fig. 15:**
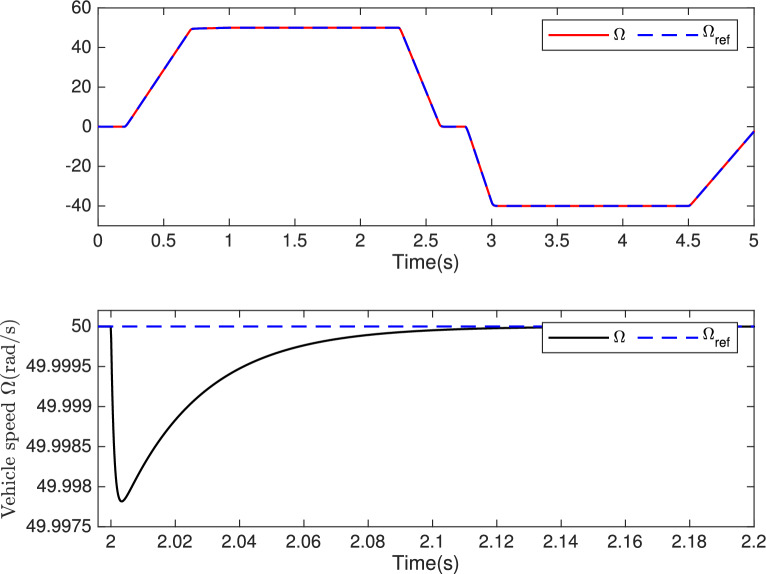
**Top:** Vehicle speed $\Omega (rad/s)$ and its reference $\Omega _{ref} (rad/s)$. **Bottom:** Zoom on vehicle speed *rad*/*s*.

**Fig. 16:**
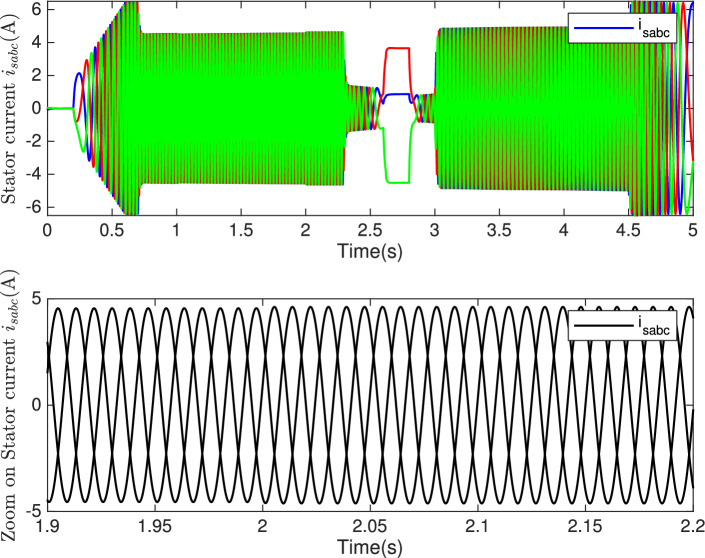
**Top:** Stator current $i_{sabc}$ (A). **Bottom:** Zoom on stator current.

To verify the robustness of the proposed control against variations in the system characteristics, the dynamic behavior of the system was studied by introducing a $30\%$ uncertainty on the nominal values of the machine parameters, including the stator resistance and inductance ($R_s, L_s$), the viscous friction coefficient ($F_m$), and the rotor inertia ($J_m$). These variations were applied to simulate realistic conditions where the machine parameters may differ from their nominal values due to wear, temperature variations, or other external factors. The performance of the proposed control was evaluated in this context, and the results are presented in Figs. 15 and 16. These figures illustrate the evolution of the machine states, including the rotor angular velocity ($\Omega$) and the stator currents in the three-phase reference frame ($I_{abc}$).

During the time interval $\left[ 2, 2.2 \right] s$ , the actual values of the machine parameters were used in the simulated model, while the nominal values were used in the control. Despite this discrepancy, after a brief transient period, the control demonstrated the ability to maintain stable and accurate performance, ensuring that the machine states converge to their reference values.

The proposed control demonstrates stability and effectiveness even under significant variations in machine parameters, confirming its robustness against system uncertainties. The results indicate that it accurately tracks speed and current references despite disturbances caused by parameter variations, as highlighted in the zoomed view in Fig. 15. Moreover, these variations do not significantly impact overall system performance, validating the robustness of the control design.

## Comparative analysis

To rigorously validate the proposed robust backstepping controller, a comparative analysis was conducted against a standard backstepping controller and a conventional PID controller, with results summarized in Table 2. All controllers were simulated in MATLAB/Simulink under identical conditions, including the reference speed profile (Figure 7) and vehicle mass variations (Figure 8). The proposed robust backstepping controller achieves a root mean square error (RMSE) of 0.12*rad*/*s*, significantly outperforming the standard backstepping controller (0.25*rad*/*s*) and PID controller (0.35*rad*/*s*), reflecting superior speed tracking precision. Its convergence time to within $2\%$ of the reference speed is 0.15*s*, compared to 0.22*s* for standard backstepping and 0.28 s for PID, indicating faster response dynamics. The peak overshoot is reduced to $1.5\%$ versus $2.8\%$ and $4.2\%$, respectively, while the steady-state error drops to 0.03*rad*/*s* from 0.07*rad*/*s* and 0.10*rad*/*s*. Moreover, under varying wind speed and mass conditions, the proposed controller maintains robust performance with RMSE values of 0.14*rad*/*s* and 0.13*rad*/*s*, compared to 0.28*rad*/*s* and 0.26*rad*/*s* for standard backstepping, and 0.40*rad*/*s* and 0.38*rad*/*s* for PID. These results, highlight the proposed approach’s enhanced accuracy, responsiveness, and resilience to parameter uncertainties.

**Table 2:** Performance comparison of proposed controller versus standard backstepping and PID controllers.

Metric	Robust backstepping	Standard backstepping	PID
RMSE (rad/s)	0.102	0.193	0.315
Convergence time (s)	0.1	0.15	0.25
Peak overshoot $\%$	1.3	2.2	3.5
Steady-state error (rad/s)	0.03	0.05	0.12
Robustness to wind speed variation (RMSE) (rad/s)	0.123	0.218	0.440
Robustness to mass variation (RMSE) (rad/s)	0.135	0.236	0.380

RMSE and steady-state error are calculated based on the difference between vehicle speed $\Omega$ and $\Omega _{ref}$.

Convergence time is the time to reach within 2% of $\Omega _{ref}$.

Peak Overshoot: Calculate as $\dfrac{\Omega _{max}-\Omega _{ref}}{\Omega _{ref} } \times 100\%$

## Conclusion

In conclusion, the primary objective of this study was to develop a precise computer-based model for Electric Vehicle (EV) energy consumption during various driving cycles. A comprehensive forward vehicle simulation model was evaluated through a simulation using MATLAB/Simulink, encompassing the intricate powertrain system and longitudinal vehicle dynamics. This model integrated the Thevenin equivalent circuit battery model, inverter, and permanent magnet synchronous motor (PMSM), contributing to a holistic roepresentation of the EV’s energy dynamics. Additionally, the resistance forces opposing the vehicle’s motion were accurately characterized within the longitudinal vehicle dynamics, further enhancing the model’s accuracy. To regulate the vehicle’s speed, a robust nonlinear controller, specifically the backstepping robust technique, was adeptly designed and incorporated into an elaborated driver model. A formal stability analysis employing Lyapunov tools provided assurance that the system operates in a stable manner, crucial for real-world applications. The results obtained from this comprehensive model undeniably demonstrate its effectiveness in achieving the defined control objectives with remarkable performance and robustness. Looking ahead, future work could focus on integrating real-time adaptive algorithms to enhance the controller’s responsiveness to unpredictable environmental conditions, such as sudden weather changes or road surface variations. Additionally, experimental validation on a physical EV prototype would help bridge the gap between simulation and practical implementation. Investigating the scalability of the proposed approach across different EV architectures (e.g., hybrid or multi-motor systems) could further broaden its applicability.

## Data Availability

The data that supports the findings of this study are available from the corresponding author on request.

## Abbreviations

CCM: Coulomb counting method
DC/AC: Direct current/Alternating current
EV: Electric vehicle
OCV: Open circuit voltage
PID: Proportional-integral-derivative
PMSM: Permanent magnet synchronous motors
RUN: Runge kutta
SOC: State of charge
RMSE: Root mean square error
MPC: Model predictive control
